# Biochemical Characterization and Elucidation of Action Pattern of a Novel Polysaccharide Lyase 6 Family Alginate Lyase from Marine Bacterium *Flammeovirga* sp. NJ-04

**DOI:** 10.3390/md17060323

**Published:** 2019-05-31

**Authors:** Qian Li, Fu Hu, Benwei Zhu, Yun Sun, Zhong Yao

**Affiliations:** College of Food Science and Light Industry, Nanjing Tech University, Nanjing 211816, China; njlq@njtech.edu.cn (Q.L.); hufu@njtech.edu.cn (F.H.); sunyun_food@njtech.edu.cn (Y.S.); yaozhong@njtech.edu.cn (Z.Y.)

**Keywords:** alginate lyase, polysaccharide lyase of family 6, characterization, degradation pattern

## Abstract

Alginate lyases have been widely used to prepare alginate oligosaccharides in food, agricultural, and medical industries. Therefore, discovering and characterizing novel alginate lyases with excellent properties has drawn increasing attention. Herein, a novel alginate lyase FsAlyPL6 of Polysaccharide Lyase (PL) 6 family is identified and biochemically characterized from *Flammeovirga* sp. NJ-04. It shows highest activity at 45 °C and could retain 50% of activity after being incubated at 45 °C for 1 h. The Thin-Layer Chromatography (TLC) and Electrospray Ionization Mass Spectrometry (ESI-MS) analysis indicates that FsAlyPL6 endolytically degrades alginate polysaccharide into oligosaccharides ranging from monosaccharides to pentasaccharides. In addition, the action pattern of the enzyme is also elucidated and the result suggests that FsAlyPL6 could recognize tetrasaccharide as the minimal substrate and cleave the glycosidic bonds between the subsites of −1 and +3. The research provides extended insights into the substrate recognition and degradation pattern of PL6 alginate lyases, which may further expand the application of alginate lyases.

## 1. Introduction

Alginate is a linear acidic polysaccharide that constitutes the cell wall of brown algae [1]. It consists of two uronic acids, namely the β-d-mannuronate (M) and the α-l-guluronate (G), which are randomly arranged into different blocks [2]. The alginate has been widely used in food, agricultural and medical industries due to its favorable properties and versatile activities. However, the applications of alginate have been greatly limited by its disadvantages such as high molecular weight, low solubility, and poor bioavailability. In addition, the alginate molecule could not get into the circulation system due to its huge molecular structure. Therefore, it could not exhibit its physiological activities. Alginate oligosaccharides, as the degrading products of alginate, are smaller with excellent solubility and bioavailability than the polysaccharides. In addition, the physiological effects, such as anticoagulant, antioxidant, and antineoplastic activities, can also be retained after degradation. Therefore, they have been widely used as anticoagulants, plant growth accelerators and tumor inhibitors in food, agricultural, and medical fields [3,4,5]. Therefore, it holds great promise to degrade the alginate to prepare functional alginate oligosaccharides [6].

Alginate lyases could degrade alginate to oligosaccharides by β-elimination mechanism and therefore they belong to the Polysaccharides Lyase (PL) family [7]. Recently, alginate lyases have drawn increasing attention for preparing alginate oligosaccharides with the advantages such as high efficiency and specificity and mild degrading conditions [8]. Up to now, numerous alginate lyases have been isolated, identified, and characterized [9]. Unfortunately, only a few show high activity and thermal stability, which are essential properties for industrial applications [10,11]. Previously, two alginate lyases with excellent characteristics have been identified from the *Flammeovirga* sp. NJ-04. In this study, a novel alginate lyase of PL 6 family has been cloned and characterized from the strain. The biochemical properties and degrading pattern of the enzyme have been investigated and this research would further expand the applications of alginate lyases in related fields.

## 2. Results and Discussion

### 2.1. Sequence Analysis of FsAlyPL6

**Figure 1:**
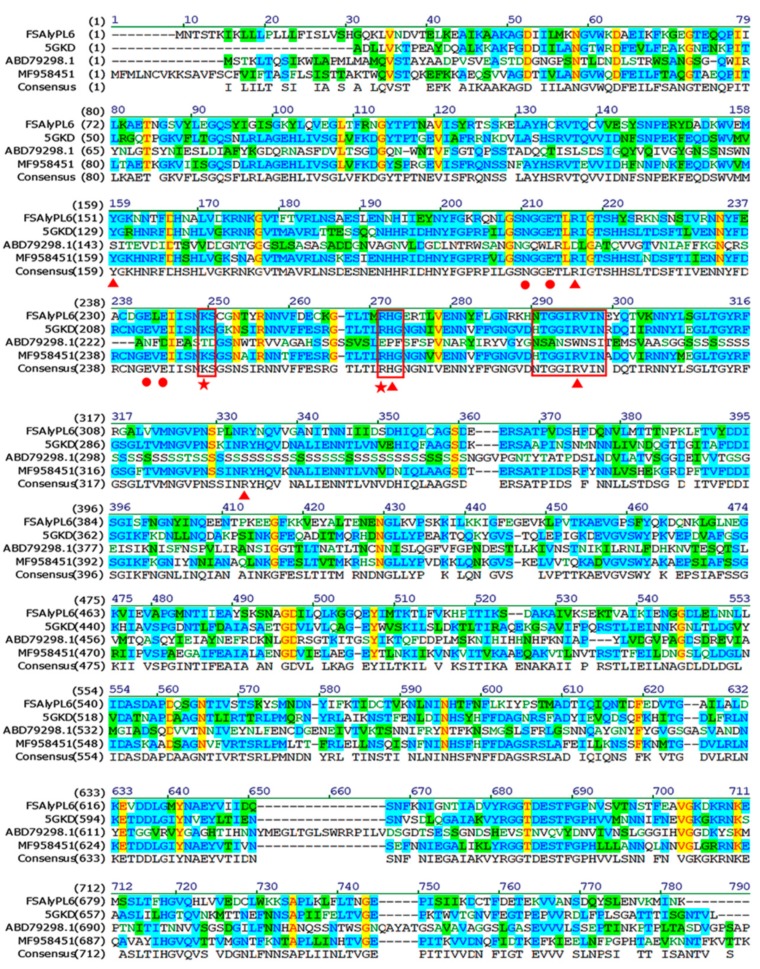
Multiple amino acid sequences alignment of AlyPL6 and other alginate lyases of PL6 family: AlyGC (BAEM00000000.1) from *Glaciecola chathamensis* S18K6T, polysaccharide lyase (ABD79298.1) from *Saccharophagus degradans* 2–40, and TsAly6A (MF958451) from *Thalassomonas* sp. LD5. Three boxes enclose conserved regions. Residues in FsAlyPL6, which are responsible for the enzymatic activity Ca^2+^ binding and catalysis, are marked in triangle, dots, and stars, respectively.

**Figure 2:**
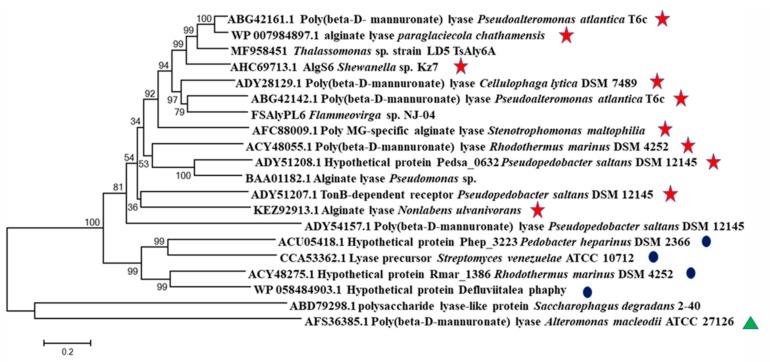
Phylogenetic analysis of FsAlyPL6 with other alginate lyases of PL6 family based on amino acid sequence comparisons. The species names are indicated along with accession numbers of corresponding alginate lyase sequences. Bootstrap values of 1000 trials are presented in the branching points. The subfamilies 1, 2, and 3 are marked with stars, dots, and triangle, respectively.

The gene of FsAlyPL6 was cloned and analyzed from *Flammeovirga* sp. NJ-04. The open reading frame (ORF) consisted of 2238 bps and encoded a putative alginate lyase of 745 amino acid residues with a theoretical molecular mass of 83.09 kDa. According to the conserved domain analysis, the FsAlyPL6 contained an N-terminal catalytic domain (Met^1^-Asn^366^) and a C-terminal domain (Gln^367^-Lys^745^). Based on the sequence alignments shown in Figure 1, FsAlyPL6 shared the highest identity (45%) with AlyGC (BAEM00000000.1) from *Glaciecola chathamensis* S18K6T, which indicated FsAlyPL6 is a new member of family PL6. In addition, FsAlyPL6 contained three conserved regions “NG(G/A)E”, “KS”, and “R(H/S)G” (marked in Figure 1), which are involved in substrate binding and catalytic activity [12]. The alginate lyases of PL6 family can be divided into three subfamilies, namely subfamilies 1, 2, and 3. In order to confirm the subfamilies of FsAlyPL6, the phylogenetic tree was used to compare the sequence homology with alginate lyases from diverse subfamilies. As is shown in Figure 2, FsAlyPL6 clustered with representative enzymes of subfamily 1, which indicated FsAlyPL6 is a new member of the subfamily 1 alginate lyase.

### 2.2. Expression and Purification of FsAlyPL6

**Figure 3:**
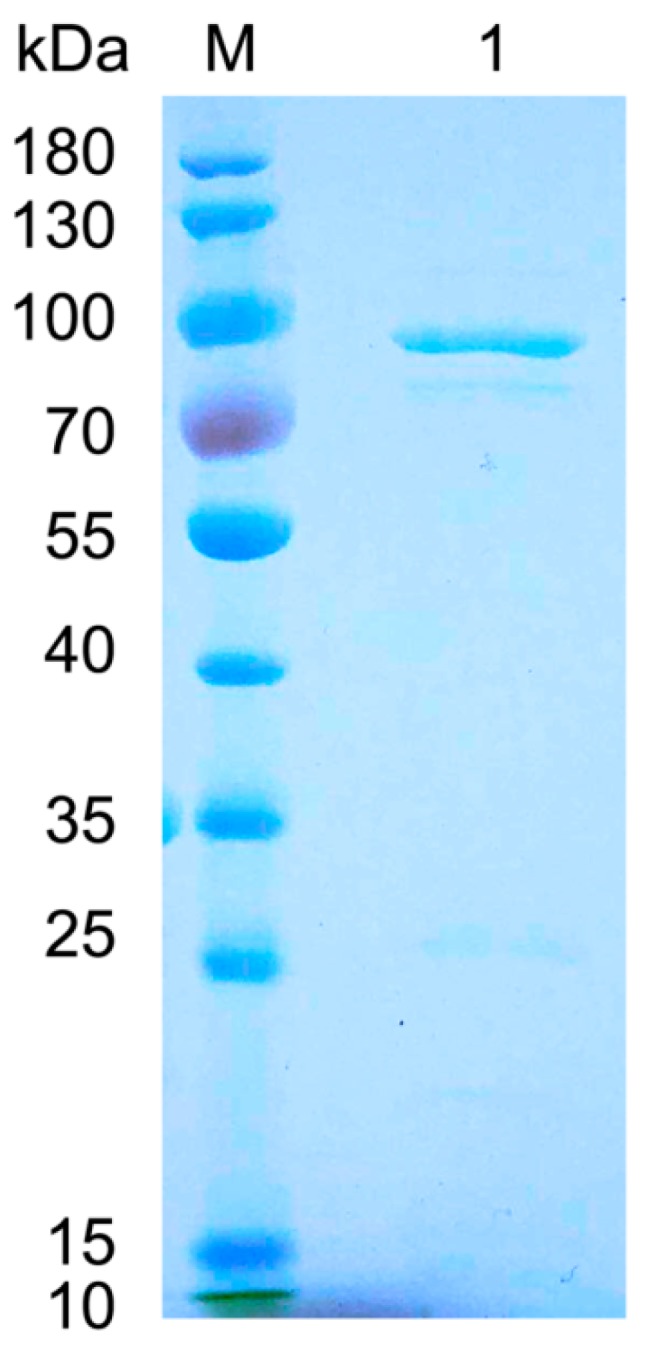
Sodium dodecyl sulfate polyacrylamide gel electrophoresis (SDS-PAGE) analysis of purified FsAlyPL6. Lane M protein: restrained marker (Thermo Scientific, Waltham, MA, USA); lane 1: purified FsAlyPL6.

The gene of FsAlyPL6 was ligated into pET-21a(+) and then the recombinant plasmid was transformed into *E. coli BL21* (DE3) for heterologously expression. The recombinant FsAlyPL6 was then purified by Ni-NTA sepharose affinity chromatography and analyzed by SDS-PAGE (Figure 3). A clear band (about 80 kDa) can be observed in gel, which was consistent with the theoretical molecular mass of 83.09 kDa. Three kinds of substrates (sodium alginate, polyM, and polyG) were employed to determine the substrate specificity of FsAlyPL6. As shown in Table 1, FsAlyPL6 exhibited higher activity towards sodium alginate (483.95 U/mg) and it showed lower activity towards to polyM (221.5 U/mg). However, it showed the lowest activity towards to polyG (19.35 U/mg). Accordingly, FsAlyPL6 is a polyMG-preferred lyase like most of PL6 family alginate lyases with the exceptions of Patl3640 from *Pseudoalteromonas atlantica* T6c and Pedsa0631 from *Pedobacter saltans* [13]. Both of them preferred polyG to polyMG blocks. In addition, TsAly6A from *Thalassomonas* sp. LD5 [14], OalS6 from *Shewanella* sp. Kz7 [15], OalC6 from *Cellulophaga* sp. SY116 [16], and AlyF from *Vibrio* sp. OU02 [17] are all characterized as polyG-preferred alginate lyases. The kinetic parameters of FsAlyPL6 towards sodium alginate, polyM, and polyG were calculated based on the hyper regression analysis. As shown in Table 1, the *K_m_* values of FsAlyPL6 towards sodium alginate, polyM, and polyG were 0.50 mg/mL, 1.52 mg/mL, and 1.62 mg/mL, respectively. FsAlyPL6 had a lower *K_m_* value towards sodium alginate than to polyM and polyG. Accordingly, FsAlyPL6 exhibited higher affinity towards MG-block than to M-block and G-block. The *k_cat_* values of FsAlyPL6 towards sodium alginate, polyM and polyG were 33.98 s^−1^, 17.66 s^−1^, and 4.98 s^−1^, respectively. It indicated that FsAlyPL6 had higher catalytic efficiency towards MG-block than to the other two blocks.

### 2.3. Biochemical Characterization of FsAlyPL6

**Figure 4:**
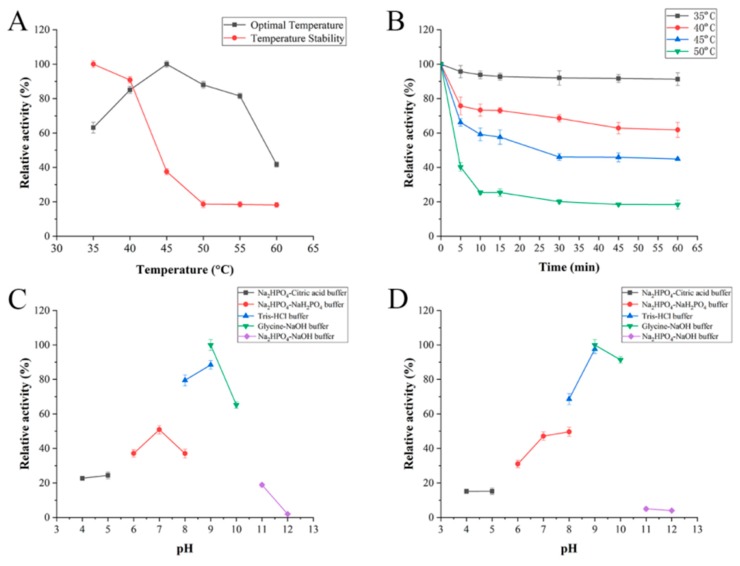
Biochemical characterization of FsAlyPL6: (**A**) The optimal temperature and thermal stability of FsAlyPL6; (**B**) the thermal-induced denaturation of FsAlyPL6; (**C**) the optimal pH of the FsAlyPL6; (**D**) the pH stability of FsAlyPL6.

The optimal temperature of FsAlyPL6 is 45 °C and it retains more than 90% of maximal activity after being incubated at 45 °C for 1 h (Figure 4A). Compared with other PL6 family alginate lyases, FsAlyPL6 exhibits preferable thermal characteristics than most PL6 family alginate lyases. For example, AlyF of *Vibrio* OU02 showed the maximal activity at 30 °C [17] and AlyGC from *G. chathamensis* S18K6T has an optimal temperature of 30 °C [12]. OalC6 of *Cellulophaga* sp. SY116 exhibits highest activity at 40 °C and retains about 80% of highest activity after being incubated at 40 °C for 1 h [16]. In addition, FsAlyPL6 retains 95% activity after being incubated at 35 °C for 60 min and inactivated gradually with temperature increased (Figure 4B). This remarkable characteristic indicated FsAlyPL6 possesses great potential in industrial applications for preparation alginate oligosaccharides. The optimal pH of FsAlyPL6 is 9.0 and it retains about 90% activity incubated at pH 9.0–10.0 for 12 h (Figure 4C,D), which indicated FsAlyPL6 is an alkaline-stable lyase. To the best of our knowledge, few alginate lyases of PL6 family are alkaline-stable lyases, and most of them exhibit the maximal activities around neutral pH values such as OalC6 of *Cellulophaga* sp. SY116 has an optimal pH of 6.6 and it retains only 60% of its maximal activity after being incubated at pH 6.0 for 6 h [16]. The OalS6 from *Shewanella* sp. Kz7 exhibits maximal activity at pH 7.2 and retains 80% after being hatched at pH 6.0–8.0 for 24 h [15]. The influences of metal ions on enzyme activity were also investigated. As shown in Table 2, like TsAly6A from *Thalassomonas* sp. LD5 [14], the activity of FsAlyPL6 can be activated by Ca^2+^ and Mg^2+^. FsAlyPL6 is inhibited by various divalent metal ions such as Cu^2+^, Zn^2+^ and Ni^2+^, which is similar to OalS6 from *Shewanella* sp. Kz7 [15].

### 2.4. Action Pattern and Substrate Docking of FsAlyPL6 Product Analysis

**Figure 5:**
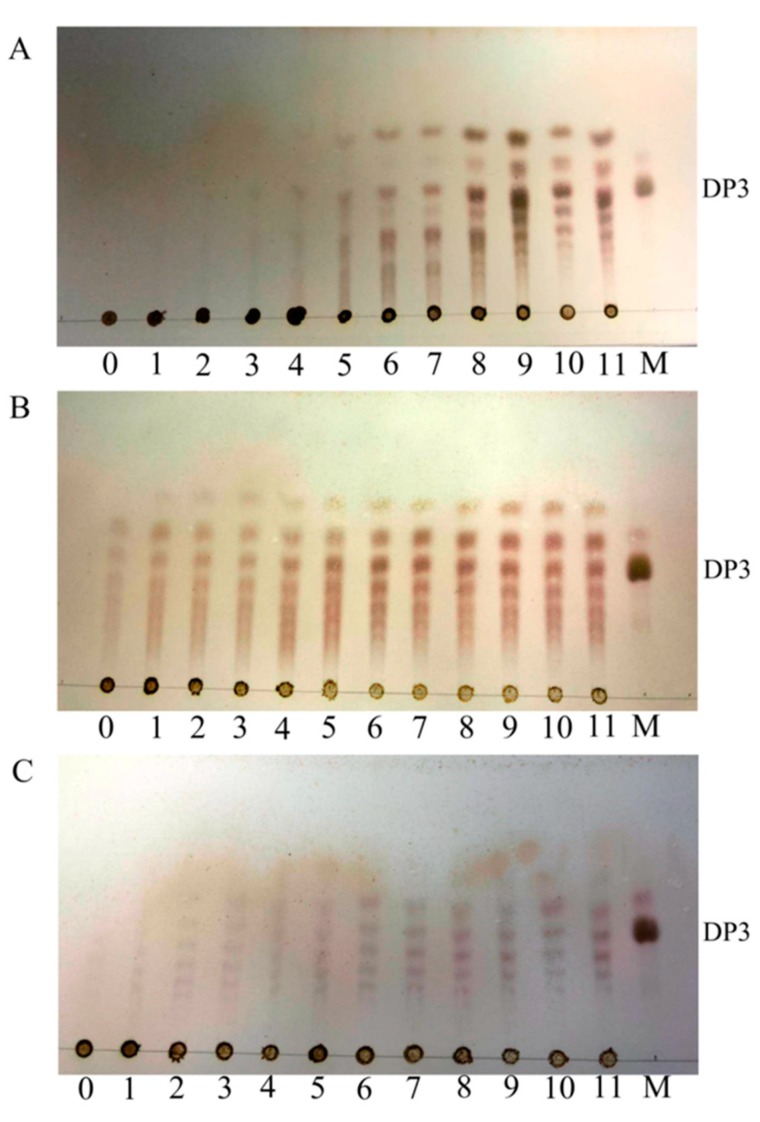
TLC analysis of degrading products of FsAlyPL6 towards alginate (**A**), polyM (**B**), and polyG (**C**). Lane M, the oligosaccharide standard; lanes 0–11, the samples taken by 0 min, 5 min, 10 min, 15 min, 30 min, 60 min, 2 h, 6 h, 12 h, 24 h, and 48 h, respectively.

**Figure 6:**
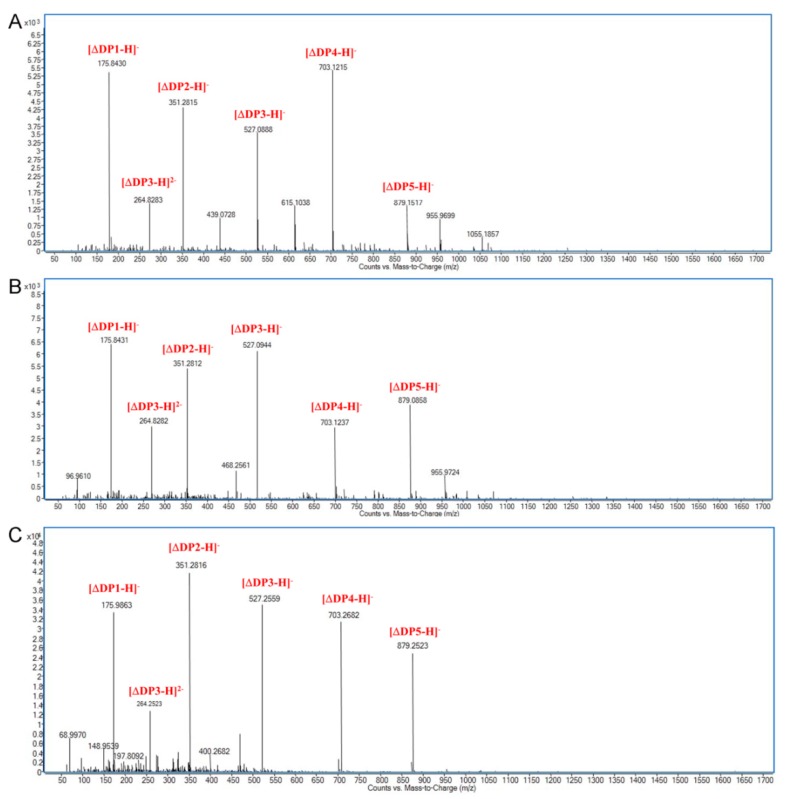
ESI-MS analysis of products of FsAlyPL6 towards alginate (**A**), polyM (**B**), and polyG (**C**).

To elucidate the action mode of FsAlyPL6, the degradation products of three substrates for different times (0–48 h) were analyzed by TLC (Figure 5). As the degrading process continues, three kinds of substrates are degraded into oligosaccharides with lower degrees of polymerization (DPs) (2–5) and monosaccharide, which indicated that FsAlyPL6 can cleave the glycosidic bonds within the substrates in an endolytic manner. The ESI-MS results indicated that degradation products of FsAlyPL6 towards the three different substrates include monosaccharide, and oligosaccharides with different DPs (2–5) can be detected (Figure 6A–C). Most of PL6 family enzymes are endo-type alginate lyases, which produce oligosaccharides with DPs (2–4). However, the Patl3640 from *Pseudoalteromonas atlantica* T6c [13], Pedsa0631 from *Pedobacter saltans* [13], OalS6 from *Shewanella* sp. Kz7 [15], and OalC6 from *Cellulophaga* sp. SY116 degrade the substrates into monosaccharides in an exolytic manner [16].

**Figure 7:**
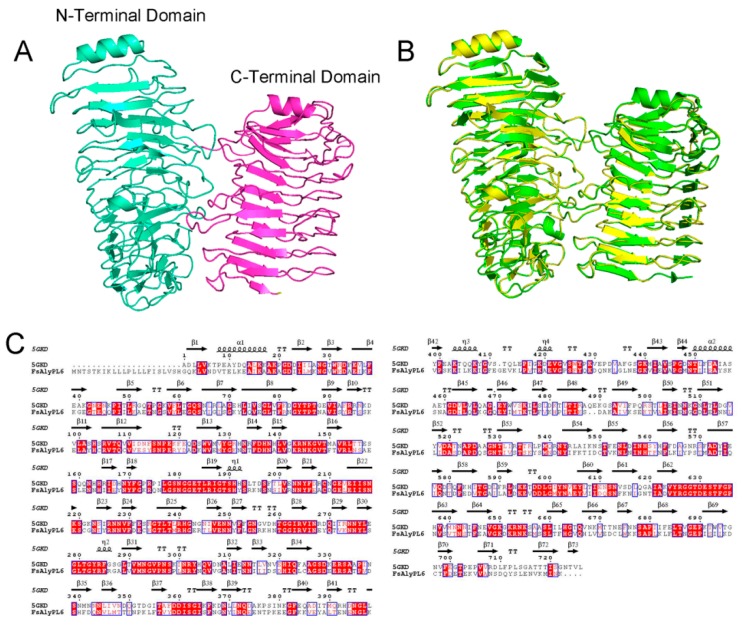
(**A**) Overall structure of FsAlyPL6; (**B**) the structural comparison of FsAlyPL6 (green) and AlyGC (yellow); (**C**) sequence alignments of FsAlyPL6 and AlyGC.

**Figure 8:**
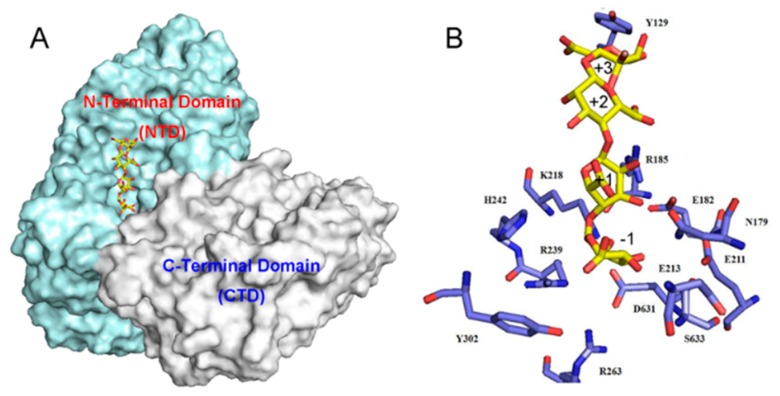
(**A**) Stereo view of the alginate tetrasaccharide (MMMM) bound to the tunnel-shaped active site of FsAlyPL6. (**B**) The presentation of catalytic residues responsible for binding and catalyzing the substrate.

The three-dimensional model of the FsAlyPL6 was constructed by PHYRE2 and the tetrasaccharide (MMMM) was docked into the FsAlyPL6. Because the sequence similarity between FsAlyPL6 and AlyGC was high (45%), the protein model was successfully constructed with 100% confidence. As shown in Figure 7A, the overall structure of FsAlyPL6 was predicted to fold into a “twin tower-like” structure (Figure 7A), which is similar to the structure of AlyGC (Figure 7B). However, AlyGC is an exo-type alginate lyase and FsAlyPL6 degrade alginate into oligosaccharide in an endolytic manner. The key residues for substrate recognition were identified by the sequence alignment and protein–substrate interactions. As shown in Figure 7C, the residues R_239_, R_263_, K_218_, E_213_, and Y_332_ are were highly conserved and involved in the interaction between the protein and substrates in subsites −1, +1, +2 and +3, respectively (Figure 8A,B). Based on the docking and β-elimination mechanism, the residues K_218_ and R_239_ acted as the Brønsted base and Brønsted acid, respectively, in the cleavage reaction of FsAlyPL6 on alginate, which is consistent with the residues of AlyGC (Figure 8B).

## 3. Materials and Methods

### 3.1. Materials and Strains

Sodium alginate (M/G ratio: 77/23) was purchased from Sigma-Aldrich (St. Louis, MO, USA). PolyG and polyM (purity: about 95%; M/G ratio: 3/97 and 97/3, respectively) were purchased from Qingdao BZ Oligo Biotech Co., Ltd. (Qingdao, China). *Flammeovirga* sp. NJ-04 was isolated from the South China Sea and conserved in our laboratory. It was cultured at 35 °C in 2216E medium (Difoc). *Escherichia coli* DH5α and *E. coli* BL21 (DE3) were used for plasmid construction and as the hosts for gene expression, respectively. These strains were cultured at 37 °C in Luria-Bertani (LB) broth or on LB broth agar plates (LB broth was supplemented with 1.5% agar and contained 100 μg/mL ampicillin).

### 3.2. Cloning and Sequence Analysis of Alginate Lyase

As previously reported, a gene cluster for degrading alginate has been identified within the genome of the strain *Flammeovirga* sp. NJ-04 [10]. According to the sequence of the putative alginate lyase gene sequence (WP_044204792.1), a pair of special primers was designed as described in Supplementary Materials. For gene expression, the alginate lyase gene *FsAlyPL6* was subcloned and then ligated into pET-21a(+) expression vector. The theoretical molecular (Mw) and isoelectric point (*p*I) were calculated using Compute pI/Mw tool (https://web.expasy.org/compute_pi/). Molecular Evolutionary Genetics Analysis (MEGA) Program version 6.0 (Center for Evolutionary Medicine and Informatics, The Biodesign Institute, Tempe, AZ, USA) was applied to construct a phylogenetic tree through a neighbor-joining method based on alginate lyase protein sequences of PL6 family. The Vector NTI (Invitrogen, Thermo Scientific, Waltham, MA, USA) was used to obtain multiple sequence alignment. The homology modeling and docking was built by Protein Homology/analogY Recognition Engine V 2.0 (Structural Bioinformatics Group, Imperial College, London, Britain).

### 3.3. Hereologous Expression and Purification of the Recombinant Enzyme

The recombinant plasmid pET-21a(+)–*FsAlyPL6* was transformed into *E. coli* BL21 (DE3). It was then cultured in an LB medium (containing100 μg/mL of ampicillin) at 37 °C by shaking at 200 rpm for 5 h, followed by being induced with 0.1 mM IPTG at 25 °C for 36 h when OD_600_ reached 0.6. The purification of FsAlyPL6 was performed as follows. The cells were harvested by centrifugation and then sonicated in lysis buffer (50 mM Tris-HCl with 300 mM NaCl, pH 8.0). The cell homogenate that contained recombinant protein were purified by using a His-trap column (GE Healthcare, Uppsala, Sweden). SDS on 12% (*w/v*) resolving gel was applied to detect the purity of the recombinant protein.

### 3.4. Substrate Specificity and Enzymatic Kinetics

The reaction was performed using 20 μL FsAlyPL6 (4 μg) mixed with 180 μL 0.8% alginate, polyM, and polyG respectively. The enzyme activity was measured using the ultraviolet absorption method [11]. One unit was defined as the amounts of enzyme required to increase absorbance at 235 nm (extinction coefficient: 6150 M^−1^·cm^−1^) by 0.1 per min. The kinetic parameters of the FsAlyPL6 towards alginate, polyM, and polyG were investigated by measuring the enzyme activity with these substrates at different concentrations (0.4–10 mg/mL). Velocity (V), *K_m_*, and *V_max_* values were calculated as previously reported [10]. The radio of *V_max_* versus enzyme concentration ([*E*]) was used to calculate the turnover number (*k*_cat_) of the enzyme.

### 3.5. Biochemical Characterization of the Recombinant Enzyme FsAlyPL6

The effects of temperature on the enzyme activity were determined by testing the activity at different temperatures (35 °C to 60 °C). The thermal stability was characterized by measuring the residual activity after the purified FsAlyPL6 was incubated at 35–60 °C for 1 h. Furthermore, the thermally induced denaturation was also determined by measuring the residual activity after incubating the enzyme at 35–50 °C for 0–60 min. To investigated the optimal pH of the FsAlyPL6, 1% alginate mixed with different buffers at 45 °C (50 mM phosphate–citrate (pH 4.0–5.0), 50 mM NaH_2_PO_4_–Na_2_HPO_4_ (pH 6.0–8.0), 50 mM Tris–HCl (pH 7.0–9.0), and glycine–NaOH (pH 9.0–12.0)) were used as the substrates and the purified enzyme incubated with these substrates under standard conditions. Moreover, the pH stability was evaluated based on the residual activity after being incubated with indifferent buffers (pH 4.0–12.0) for 20 h. The effects of metal ions on the enzymatic activity were performed by incubating the FsAlyPL6 with substrates that contained various metal compounds with a final concentration of 1 mM. The reaction performed under standard tested conditions and the substrates blend without any metal ion was taken as the control.

### 3.6. Action Pattern and Degradation Product Analysis

In order to elucidate the action pattern of the FsAlyPL6, the thin-layer chromatography (TLC) was applied to analyze the degrading products of FsAlyPL6 towards sodium alginate, polyM and polyG. The reaction and treatment of the samples were performed as previously reported [10]. In order to investigate the composition of the degrading products, ESI-MS was employed as follows: The supernatant (2 μL) was loop-injected to an LTQ XL linear ion trap mass spectrometer (Thermo Fisher Scientific, Waltham, MA, USA) after centrifugation. The oligosaccharides were detected in a negative-ion mode using the following settings: ion source voltage, 4.5 kV; capillary temperature, 275–300 °C; tube lens, 250 V; sheath gas, 30 arbitrary units (AU); and scanning the mass range, 150–2000 *m/z*.

### 3.7. Molecular Modeling and Docking Analysis

Protein Homology/analogY Recognition Engine V 2.0 was applied to construct the three-dimensional structure of FsAlyPL6 according to the known structure of alginate lyase AlyGC from *Glaciecola chathamensis* S18K6T (PDB: 5GKD) with a sequence identity of 45%. The molecular docking of the FsAlyPL6 and MMMM was performed using Molecular Operating Environment (MOE, Chemical Computing Group Inc., Montreal, QC, Canada). The ligand-binding sites were defined using the bound ligand in the homology models. PyMOL (http://www.pymol.org) was used to visualize and analyze the modeled structure and to construct graphical presentations and illustrative figures.

## 4. Conclusions

In this study, we reported a new PL family alginate lyase FsAlyPL6 from the marine *Flammeovirga* sp. NJ-04. It preferred to degrade the polyMG block and showed highest activity at 45 °C and could retain 50% of activity after being incubated at 45 °C for 1 h. The FsAlyPL6 endolytically degraded alginate polysaccharide and released oligosaccharides with DPs of 1–5. In addition, it could recognize tetrasaccharide as the minimal substrate and cleave the glycosidic bonds between the subsites of −1 and +3 to release oligosaccharides. The research provides extended insights into the degradation pattern of PL6 alginate lyases and further expands the application of alginate lyases.

## Figures and Tables

**Table 1:** Specificity and kinetics of FsAlyPL6.

Substrate	Sodium Alginate	PolyM	PolyG
Activity (U/mg)	483.95	221.5	19.35
*K_m_* (mg/mL)	0.50	1.52	1.62
*V_max_* (nmol/s)	1.36	0.71	0.20
*k_cat_* (s^−1^)	33.98	17.66	4.98
*k_cat_*/*K_m_* (mL·s^−1^·mg^−1^)	62.91	11.58	3.08

**Table 2:** Effects of metal ions on activity of FsAlyPL6.

Reagent	Relative Activity (%)
Control	100.00 ± 2.97
K^+^	93.26 ± 2.23
Na^+^	118.57 ± 1.08
Ca^2+^	104.33 ± 1.12
Mg^2+^	102.31 ± 2.78
Co^2+^	22.14 ± 1.32
Zn^2+^	24.88 ± 3.57
Cu^2+^	15.28 ± 1.20
Ni^2+^	50.19 ± 3.93
Mn^2+^	6.46 ± 0.60
Fe^3+^	26.55 ± 1.21

## Supplementary Materials

The following are available online at https://www.mdpi.com/1660-3397/17/6/323/s1, Table S1: The primers for cloning the gene of FsAlyPL6.
