# Supplementary material for: Biochemical Characterization and Elucidation of Action Pattern of a Novel Polysaccharide Lyase 6 Family Alginate Lyase from Marine Bacterium Flammeovirga sp. NJ-04
Source: Mar Drugs. 2019 May 31;17(6):323. doi: 10.3390/md17060323 (PMC6627919; doi:10.3390/md17060323)
Supplement: Supplementary file 1 [file marinedrugs-17-00323-s001.pdf]

Table S1 The primers for cloning the gene of FsAlyPL6.

FsAlyPL6F	5′- CATATGCAAAAGCTTGTGAACGATGT-3′
FsAlyPL6R	5′-CTCGAG CTTATTGATCATTTTCACGT-3′
